# Rheological and Mechanical Properties of Thermoplastic Crystallizable Polyimide-Based Nanocomposites Filled with Carbon Nanotubes: Computer Simulations and Experiments

**DOI:** 10.3390/polym14153154

**Published:** 2022-08-02

**Authors:** Victor M. Nazarychev, Gleb V. Vaganov, Sergey V. Larin, Andrey L. Didenko, Vladimir Yu. Elokhovskiy, Valentin M. Svetlichnyi, Vladimir E. Yudin, Sergey V. Lyulin

**Affiliations:** Institute of Macromolecular Compounds, Russian Academy of Sciences (IMC RAS), Bolshoi pr. 31 (V.O.), 199004 St. Petersburg, Russia; nazarychev@imc.macro.ru (V.M.N.); glebvaganov@mail.ru (G.V.V.); vanilin72@yandex.ru (A.L.D.); vlad_elokhovskiy@bk.ru (V.Y.E.); valsvet@hq.macro.ru (V.M.S.); yudin@hq.macro.ru (V.E.Y.); s.v.lyulin@gmail.com (S.V.L.)

**Keywords:** polyimides, single-walled carbon nanotubes, polymer nanocomposites, crystallization, rheological properties, mechanical properties

## Abstract

Recently, a strong structural ordering of thermoplastic semi-crystalline polyimides near single-walled carbon nanotubes (SWCNTs) was found that can enhance their mechanical properties. In this study, a comparative analysis of the results of microsecond-scale all-atom computer simulations and experimental measurements of thermoplastic semi-crystalline polyimide R-BAPB synthesized on the basis of dianhydride R (1,3-bis-(3′,4-dicarboxyphenoxy) benzene) and diamine BAPB (4,4′-bis-(4″-aminophenoxy) biphenyl) near the SWCNTs on the rheological properties of nanocomposites was performed. We observe the viscosity increase in the SWCNT-filled R-BAPB in the melt state both in computer simulations and experiments. For the first time, it is proven by computer simulation that this viscosity change is related to the structural ordering of the R-BAPB in the vicinity of SWCNT but not to the formation of interchain linkage. Additionally, strong anisotropy of the rheological properties of the R-BAPB near the SWCNT surface was detected due to the polyimide chain orientation. The increase in the viscosity of the polymer in the viscous-flow state and an increase in the values of the mechanical characteristics (Young’s modulus and yield peak) of the SWCNT-R-BAPB nanocomposites in the glassy state are stronger in the directions along the ordering of polymer chains close to the carbon nanofiller surface. Thus, the new experimental data obtained on the R-BAPB-based nanocomposites filled with SWCNT, being extensively compared with simulation results, confirm the idea of the influence of macromolecular ordering near the carbon nanotube on the mechanical characteristics of the composite material.

## 1. Introduction

The development of nanocomposite materials with enhanced properties by the addition of various types of nanoparticles to polymer matrices is one of the most dynamically growing areas of polymer material science [1,2]. The primary interest in the choice of nanofillers is focused on anisometric objects of cylindrical and lamellar shape with a high aspect ratio as carbon nanofillers (nanotubes or graphene [3,4]), also as high-aspect-ratio biomaterials (fiber-reinforced composites (FRCs) [5] and cellulose nanocrystals (CNCs) [6]), that have a prospective use in the fabrication of new materials for dentistry [7] and food packaging [8]. The use of such fillers, even at low concentrations (several percent), creates a significant modification of the mechanical properties compared to that of the unfilled polymer matrix. Among nanoobjects, carbon nanoparticles gained the greatest interest in modifying the properties of polymer materials [9]. These nanofillers have excellent mechanical, thermophysical, electrical properties, low molecular weight, and resistance to chemical solvents [10,11]. Additionally, the addition of one-dimensional fillers such as carbon nanotubes might initiate the structural ordering of different polymers close to their surfaces such as polyethylene [12,13,14,15,16], polypropylene [17,18,19], polyvinyl alcohol [20], polylactic acid [21], polyamides [13,22], polyetheretherketone [23,24,25], heterocyclic polyalkylthiophenes [26] and aromatic polyimides [27,28,29,30]. In general, polymer matrix ordering in nanocomposites filled with carbon nanotubes leads to improvement of the nanocomposite performance properties.

Thermally stable aromatic polyimides (PI)s deserve particular attention as matrices in the production of nanocomposites [31,32]. Incorporating carbon nanoparticles could influence changes in the mechanical and other properties of PIs [33,34,35,36]. For instance, in our previous study [8], it was experimentally shown that the addition of 1% mass of vapor-grown carbon fiber (VGCF) and 0.1% single-walled carbon nanotube (SWCNT) leads to improved mechanical properties of PI fibers derived from the commercially available amorphous polyetherimide Ultem 1000. The enhancement of the thermophysical and mechanical characteristics of nanocomposites based on thermoplastic PIs was shown both in the experimental study and computer simulation [27,28,37,38,39,40,41,42,43]. The reinforcement of polymers by carbon nanoparticles can lead to the initialization and acceleration of the crystal growth of PIs [27,28,39,40,41,42,43].

Among the group of aromatic PIs, the new crystallizable PIs are characterized by higher heat and chemical resistance and the enhanced mechanical properties of the products derived from them [44,45,46]. PI R-BAPB developed in the IMC RAS is one of the striking representative PIs capable of crystallization [47,48]. This thermoplastic PI is synthesized on the basis of dianhydride R (1,3-bis-(3′,4-dicarboxyphenoxy) benzene) and diamine BAPB (4,4′-bis-(4″-aminophenoxy) biphenyl). The crystallization and recrystallization of R-BAPB were shown for films, fibers, and binders of composite materials [37,47,49]. That change in the spatial structure of the PI reduces the water wettability [50] of crystallizable PI R-BAPB; therefore, it might be also considered a promising material to produce water barrier composites. Additionally, the relatively low melting point (593 K) and low melt viscosity (up to 1000 Pa·s) allow R-BAPB processing through injection molding, extrusion, and hot pressing and for use in FDM 3D printing. 

Nevertheless, it is important to control the viscosity of both unfilled PIs and nanocomposites to process these materials in the melt. First of all, melt viscosity is determined by the chemical structure of PI, and introducing hinge groups in the chemical structure of a PI repeating unit makes it possible to control their melt viscosity [51,52], as well as the temperature range at which the carbon nanofiller could be introduced into the polymer matrix. At the same time incorporating carbon nanoparticles in a PI binder leads to a sharp increase in viscosity at low strain rates and a decrease in viscosity at higher strain rates (shear thinning behavior) [47,53]. In previous experimental studies, this effect is typically explained by the formation of a percolation mesh between carbon nanoparticles [54,55,56], rather than by the orientation of the R-BAPB chains near the surface of the nanoparticles. As an increase in PI melt viscosity should be avoided or at least controlled for practical purposes, one should know what the actual reasons for viscosity change are during melt processing. The investigation of this problem is the main goal of our work.

In our previous studies [39,40], a strong orientation of R-BAPB chain fragments was found in the vicinity of carbon nanotubes using computer simulation, which can be considered the initial stage of this PI crystallization. However, the question arises whether nanoparticle-induced polymer chain orientation in the melt could cause the viscosity change instead of the formation of interchain links. Furthermore, the change in viscosity based on the orientation of the carbon nanotubes and polymer matrix might be particularly essential once the material is cooled down to regulate the anisotropy of mechanical characteristics at room temperature [39,40,42,43]. It is complicated to directly determine this in the experiment because it is practically impossible to measure the rheological properties of the polymer interface close to the nanofiller in the directions along and across the surface of carbon nanoparticles. Thus, computer simulation could provide a way to shed a light on this problem. 

Using computer simulation in our previous study [41], the effect of the structural ordering of PI near the graphene surface on the mechanical properties of the R-BAPB was investigated. It was shown that the R-BAPB interface layer on the surface of graphene exhibits anisotropy of mechanical properties (the mechanical characteristics increase in the graphene surface and decrease perpendicularly to graphene) and an increase in the average values of the elastic modulus and the yield stress compared to the values of these characteristics of an amorphous (nonoriented) sample was shown [41]. These results for graphene are in good agreement with our other studies [42,43], in which we found the orientation of the crystallizable PIs of BPDA-P3 and ODPA-P3 near the SWCNT surface, which causes the enhancement of mechanical properties of the nanocomposites. However, due to the fact that the carbon nanotube axis in these studies [42,43] is arbitrarily located with respect to the deformation axes due to the rather small nanotube length, the study in the simulation of the anisotropy of the mechanical properties of the polymer near the SWCNT surface has not yet been performed. There is a lack of understanding of the correlation between the dependence of the rheological properties of the polyimide chain on the direction of application of shear deformation with both a change in structural ordering near the SWCNT in the melt and a change in their mechanical properties in the glassy state. Additionally, in our previous studies [39,40,42,43], we considered an SWCNT of a relatively small length (around 5 nm only), which is much smaller than in the experiment, where the length varied from units to thousands of nanometers [57]. 

From a general point of view, the longer SWCNTs might also induce stronger polymer structural ordering near their surface than smaller nanotubes [58,59]. Additionally, because of the lack of edges in the infinite nanotube, only the influence of the nanotube surface, which determines the ordering of the crystallizable polymer near its surface, can be investigated in the simulation and the appearance of percolation barriers between the nanofiller particles can be excluded. In particular, to investigate the mechanical properties of polymer nanocomposites, a longer SWCNT embedded in the polymer binder might give the opportunity to more precisely predict the experimental deformation behavior of polymer nanocomposites in computer simulation compared to the simulation in which the nanotubes with a shorter length were used. Thus, taking into account the long SWCNT might be more realistic for simulated mechanical and rheological deformations of the polymer nanocomposites as more closely reproduced experimental effects depending on the orientation and length of SWCNTs. Finally, computer simulations of the polyimide nanocomposites with an infinite-long carbon nanotube (which can be easily modeled as a result of periodic boundary conditions without a significant change in the number of atoms in the simulation box) oriented in the deformation direction give an opportunity to study the anisotropy of the performance properties.

Thus, in this work, the effect of the change in the supramolecular structure of R-BAPB in the vicinity of finite-length SWCNT previously found in the simulation [39,40] on the rheological properties of this PI with the addition of carbon nanofiller will be performed using an experimental study and computer simulation. It should be noted that previous studies mainly compared the mechanical properties of polymer nanocomposites with the experimental data [60,61] but not the rheological properties.

In our previous work [62], the correlation between the slow down of the PI dynamic properties in the melt and the enhancement of the mechanical properties in the glassy state was found. In the current study, we further continue to study the change of rheological properties of crystallizable PI in the melt that correlate with the change in its mechanical properties in the glassy state. The use of infinite-long carbon nanotubes may allow for the study of near-experimental-length carbon nanotubes using computer simulations, which will lead to a more accurate study of the structural ordering arising near the surface of long nanotubes on the mechanical and rheological properties of polyimide-based nanocomposites. In the simulation, the influence of the PI chain’s orientation near the SWCNT surface on the change of viscosity of the SWCNT-filled PI in the melt state will be investigated and the anisotropy of the rheological and mechanical properties of the PI chains ordered by infinite SWCNT will be studied. The results of computer simulation of polymer nanocomposites with embedding the infinite-long SWCNT for the modeling of the mechanical properties will be compared with experimental data. Such a detailed comparison of computer simulations and experimental measurements for rheological and mechanical properties using polymers with complex chemical composition, considering their structural ordering in the vicinity of carbon nanofiller, has not been carried out yet.

### Object of Study

Similar to our previous study [25], dianhydride 1,3-bis(3′,4-dicarboxyphenoxy) benzene (dianhydride R) with a melting temperature of (*T_m_*)~436–438 K, TechChemProm Ltd. (Yaroslavl, Russia), and diamine 4,4-bis(aminophenoxy) biphenyl (BAPB), *T_m_*~471–472 K, VWR International (Radnor, PA, USA), were used as monomers for synthesis of R-BAPB polyimide. Phthalic anhydride was chosen as a chain growth-limiting agent for polycondensation, *T_m_*~404–407 K, Sigma-Aldrich Co. LLC (St. Louis, MO, USA). Triethylamine, benzene, and acetic anhydride were obtained by Sigma-Aldrich Co. LLC. SWCNTs with a diameter of 1.5 nm and a length of 1–5 μm (Carbon ChG LLC, Chrenogolovka, Russia) were used to modify PI R-BAPB.

**Figure 1:**
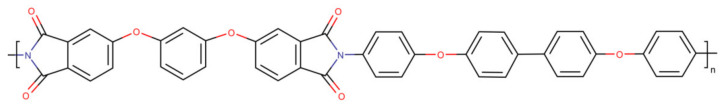
The chemical structure of the considered thermoplastic R-BAPB repeating unit.

The method of chemical imidization was used to synthesize the R-BAPB powder. The first stage of the synthesis included the polycondensation of dianhydride R with diamine BAPB into polyamic acid (PAA) in a solution of an amide solvent N-methylpyrrolidone (N-MP). Then, the chain growth process was terminated by introducing phthalic anhydride into the reaction mixture for the chemical deactivation of the terminal groups of the macromolecule. A suspension of SWCNT in N-MP was introduced in the PAA solution. Additionally, the chemical cyclization of PAA was performed according to the scheme described by us in an earlier study [48]. The synthesis resulted in a fine powder of R-BAPB (the chemical structure is shown in Figure 1) and its nanocomposites. 

## 2. Experimental and Theoretical Methods

### 2.1. Experimental Techniques

Polyimide fibers were obtained by the melt method from the R-BAPB powder modified with SWCNT using a twin-screw micro extruder from DSM Xplore (Sittard, The Netherlands) with a special fiber production machine (DSM Film Device Machine). The synthesized R-BAPB powder was loaded into the micro extruder heated to 633 K. The melt was mixed at a temperature of 633 K for 5 min to remove air and made homogeneous in the melt at a screw rotation speed of 50 rpm. The fiber was formed at a screw rotation speed of 25 rpm using a round die with a diameter of 1 mm at the exit from the extruder. At the exit from the spinneret, the fiber was cooled by a stream of air and wound at a constant speed onto a receiving coil. As a result, a monofiber was formed on the basis of the R-BAPB polymer. 

We performed the investigation of the mechanical properties of the PI fibers on a universal tensile testing machine Instron 5940 (USA) with a base length of 30 mm and a loading rate of 10 mm/min. Based on the test results, the following characteristics were determined: Young’s modulus (*E*), tensile strength (*σ*), and deformation at break (*ε*). Young’s modulus was calculated as the tangent of the inclination angle of the linear fit of the stress-strain dependence in the reversible elasticity region (~0.5% strain). The yield point *σ_y_* was determined as the maximum value of the stress at which there is a deviation from the linear dependence *σ*(*ε*). The stress-strain curves were calculated by averaging the five independent samples.

The viscosity of the R-BAPB melt filled by SWCNTs was measured on an MCR-301 rheometric system (Anton Paar, Austria) using a cone-plane pair. We performed the test at a temperature of 633 K in an oscillating mode with a frequency of 1 rad/s.

### 2.2. Computer Simulations: Model and Methods 

The microsecond-scale molecular dynamics simulations were carried out by Gromacs v. 5.0.5 software (KTH-Royal Institute of Technology, Stockholm, Sweden) [63,64] using Gromos 53a5 force field [65]. 

To generate an initial configuration of the studied sample, we used the methodology developed by us earlier [39,40], namely, 27 partially folded polymer chains of R-BAPB with a polymerization degree *N_p_* = 8 were randomly inserted into a periodic simulation box at T = 600 K with an SWCNT of (5,5) chirality and diameter 0.7 nm. The polymerization degree used corresponds to the beginning of the “polymer mode” in the Fox-Flory dependence of the glass transition temperature on the molecular weight of PI with a similar chemical structure [66]. We should emphasize that in our previous studies [39,40,42,43], the structural and mechanical properties of PI-based nanocomposites comprised short-length SWCNTs randomly embedded in the polymer binder were investigated (SWCNT of length 4.7 nm was placed in a cubic cell with 6 nm-long edges). However, in experimental studies, typically the SWCNTs have a larger length from a few tens of nanometers up to several centimeters [57,67] and characterized by a higher surface area that increases the interaction with polymer samples and may sufficiently influence the structural ordering of polymer chains near the carbon nanotube and eventually affect the performance (mechanical and rheological) properties of the polymer nanocomposites.

**Figure 2:**
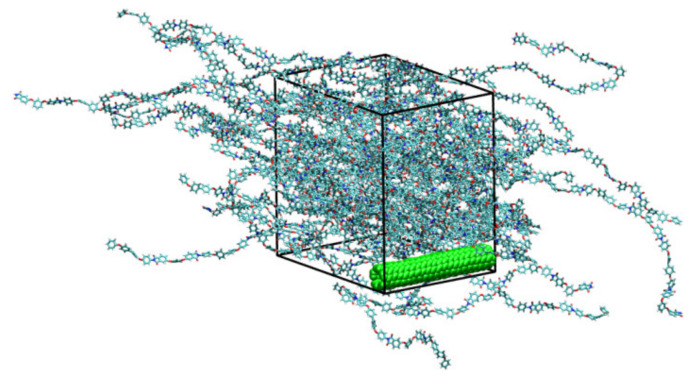
Instant configuration of the SWCNT-R-BAPB nanocomposite after 10 μs-long simulations. The SWCNT’s atom is shown in green on a snapshot. The nanotube is periodic and passes through all periodic images of a simulation box. We generated the snapshot using VMD package [69].

In this study, to eliminate the SWCNT edge effect and more accurately reproduce the experimental length of nanotubes in the simulation, the infinite-long SWCNT is considered. We applied the periodic boundary conditions in all directions of the simulation box. The SWCNT is directed along the Z-axis and, thus, in this direction, it is periodic, Figure 2. The length of the SWCNT periodic image and, therefore, simulation box in the direction of Z-axis was 4.6 nm. Because of the relatively low polarizability of R-BAPB considering partial charges has a weak impact on the PI structural properties [39,40]. Thus, to accelerate the polymer translational mobility, the partial charges are taken to be equal to zero both for PI and SWCNT. The P-LINCS algorithm [68] was applied to keep constant length for all bonds.

The mass fraction of SWCNT in the simulation box is approximately 3%. Although this value is higher than mass fraction of the filler in experimental part, the results obtained in simulation could be compared with experimental ones because concentration of the filler in experiment is below the percolation threshold and percolation of nanotube in simulation box is avoided by design.

Taking into account periodic boundary conditions we could consider this case as an array of long parallel SWCNTs with a constant distance between neighboring nanotubes equal to the simulation box dimensions. So, in our setup, we could change the concentration of SWCNT (and distance between nanotubes) by changing simulation box size which can be realized by change of the number of PI chains (or PI mass fraction) in the system considered. In this case, a decrease in PI mass fraction will lead to a decrease in the distance between SWCNTs or effective increase in the number of nanotubes in the same simulation volume. We suppose that there is some critical point where the SWCNTs will be located too close to each other and spatial constraints due to dense SWCNTs packing will prevent PI chains ordering and, probably, lead to decrease in mechanical and rheological properties of a composite. This conclusion is also supported by our preliminary not published yet results. Unfortunately, to decrease SWCNT concentration in simulation box to experimental value we need to tremendously increase amount of polymer in a box which would make simulation work extremely resource-intensive and impossible from a practical point of view. To conduct the 10 µs-long all-atom MD simulation for a system size considering 64 processors, took almost a year of continuous simulation. The increase in the number of PI atoms by 3 times leads to a slow-down in the computer performance and leads to the necessity of simulation for a few years.

After generation of the initial composite configuration, compression of the system was performed in the X and Y directions of the system at a temperature of 600 K, which is about 100 degrees higher than the glass transition temperature (~470 K [70]) of the PI R-BAPB. The overall compression time was approximately ~10 ns during which the pressure was gradually increased to 300 bar. Upon compression, the density of the system reached a constant value close to the experimental one. After that, the pressure was immediately reduced to 1 bar. Nanocomposite samples based on R-BAPB with the addition of SWCNT were generated using an isobaric-isothermal (NPT) ensemble with the help of a similar procedure [66]. To maintain constant temperature and pressure, we used a Berendsen thermostat and barostat with the time constants *τ_t_* and *τ_p_* equal to 0.1 ps and 0.5 ps, correspondingly [39,40]. 

To test the effect of structural ordering near the SWCNT surface on the rheological and mechanical properties of R-BAPB nanocomposites, a 10 μs-long simulation was carried out. During this simulation, the average sizes (end-to-end distance and radius of gyration) of the polymer chains reached their constant values in the melt [71], see Figure S1 in the Supplementary Information. 

The last 700 ns of the 10 μs-long simulation were chosen as a production run to investigate the properties of R-BAPB-based nanocomposites. In the first stage, seven configurations, separated by a time interval of 100 ns (from 9.3 to 10.0 μs), were saved. As shown earlier, a time of 100 ns is sufficient for density relaxation in the systems studied, therefore, initial configurations chosen this way might be considered as independent [71]. Then, these seven samples were cooled down to room temperature (290 K, this condition guarantees the glassy state of the considered systems) with a cooling rate γ_c_ = 1.5 × 10^11^ K/min, which was earlier used to study the mechanical properties of thermoplastic PIs in a glassy state [72] and is standard in molecular dynamics simulations for other polymers [73,74]. During the cooling procedure, we saved nanocomposite configurations at different temperatures. Then, the carbon nanofiller was removed from these samples by deleting coordinates of SWCNT atoms from the system configuration file and corresponding changes in topology file. Then, 1 ns-long simulation was performed to fill the cavity formed by the removal of SWCNT, similar to the procedure suggested by us previously for graphene-reinforced [41] nanocomposites. 

The removal of SWCNT gives the opportunity to study the rheological and mechanical properties only of the polymer chains oriented close to the nanofiller surface and eliminates the influence of the nanofiller structure on the calculated properties. We should note that 1 ns of simulation was all that was required in order to fill the cavity formed upon SWCNT removal in the melt state. In the glassy state, in addition to the 1 ns compression, in order to fill the cavity in the system formed upon removal of SWCNT, a 20 ns-long simulation was carried out with a rather high constant pressure of 50 bar applied along the *X*-axis. Such a high compression allows us to fill a cavity during simulation times lower than the relaxation times of polymer chains at simulated temperature T = 290 K and, therefore, investigate the effect of polymer chain orientation induced by SWCNT on the properties of the system. After compression, the pressure was reduced to 1 bar and an additional 1 ns-long run was performed [41]. Next, cyclic shear (in the melt state) and uniaxial deformation (in the glassy state) of the R-BAPB samples were carried out to study the rheological and mechanical properties, correspondingly.

The rheological properties of PI R-BAPB were investigated using cyclic shear deformation of the polymer melt [75], during which the volume of the R-BAPB sample was kept constant using an isochoric-isothermal ensemble (NVT). The value of shear stress *σ_xy_* upon applying shear deformation in the XY direction was calculated as -*P_xy_*, where *P_xy_* is a non-diagonal component of the pressure tensor. We calculated the shear strain as the change in a tangent of a tilt angle of the simulation box in the shear direction. The 10 oscillation cycles of the shear deformation were performed. The time dependences, which were averaged over 10 oscillation cycles of shear stress, were approximated using the formalism of large-amplitude oscillation shear (LAOS) to calculate the rheological characteristics [76,77].

(1) $$\sigma \left(t\right)=\sum_{n,odd}^{11}\gamma_{\max}^{n}\left[G_{n}^{\prime }\sin\left(n\omega t\right)+G_{n}^{\prime \prime }\cos\left(n\omega t\right)\right],$$

where $\gamma_{max}^{n}$ is the amplitude at deformation for the *n*-th harmonic, $G_{n}^{\prime }$ and $G_{n}^{\prime \prime }$ are the elasticity and loss moduli, correspondingly, ω is the cyclic strain rate, and *n* is the harmonic number [75]. The complex viscosity *η*^∗^ value is determined using the following equation:

(2) $$\eta^{*}=\sqrt{{\left(G^{\prime }/\omega \right)}^{2}+{\left(G^{\prime \prime }/\omega \right)}^{2}}$$

The frequency ω = 1.74 × 10^11^ Hz and amplitudes $\gamma_{\max}^{1}=0.12$ of cyclic deformation were chosen to study the rheological properties of thermoplastic PI R-BAPB ordered near SWCNT surface [75]. 

The effect of structural ordering on the mechanical properties of R-BAPB was investigated. As in our previous work [41], uniaxial deformation of the R-BAPB samples was carried out along X, Y, or Z coordinate axes with strain rate *γ_d_* = 1.8 × 10^8^ s^−1^. During strain procedure, the stress-strain dependences *σ*(*ε*) were calculated and analyzed further [72]. The uniaxial strain procedure comprised an affine change of atom coordinates with a constant rate along one direction of the coordinate axes (X, Y, or Z).

The values of the pressure tensor *P_i_*, where *i* = {*x*, *y*, *z*}, and the dimensions of the simulation box *L_i_* in the direction of elongation were saved to calculate the mechanical characteristics. We calculated them as the dependence of stress *σ* on strain *ε* using the following relations [78]:

(3) $$\begin{array}{l} \sigma =-P_{i},\\ \varepsilon =\frac{L_{i}-L_{0i}}{L_{0i}}, \end{array}$$

where *L*_0*i*_ is the size of the simulation box before deformation starts (*t* = 0).

The initial part of the dependence *σ*(*ε*) has a pronounced linear form corresponding to the region of linear viscoelasticity [78]:

(4) σ=Eε,

where *E* is Young’s modulus. 

We calculated the value of *E* as the slope of the linear fitting of the dependence *σ*(*ε*) in the region of linear viscoelasticity (up to 2% of strain) [72]. The value of the yield stress *σ_y_* was determined as the maximum on the dependence *σ*(*ε*), Equation (4). 

## 3. Results and Discussion

We should note that strong structural ordering of R-BAPB polymer chains in the vicinity of infinite-long SWCNT was observed. To investigate the structural properties of PI in the vicinity of the SWCNT, surface analysis of nematic ordering parameter S_N_ [79,80,81] for the PI chains and orientation of flat fragments of PI monomer units relative to the SWCNT axis was performed (see Section S2 in the Supplementary Information). The results obtained show that the increase in PI chains ordering occurs during the first 5 ms of simulation and after this time S_N_ fluctuates around an average value of ~0.5 (Figure S2 in the Supplementary Information). At the same time, the flat fragments of the R-BAPB repeating unit (Figure S3 in the Supplementary Information) near the nanofiller surface are mainly oriented along the nanoparticle surface (Figures S4 and S5 in the Supplementary Information). Thus, as in our previous studies [39,40,41,42,43], the structural ordering of the polymer chains was shown in the vicinity of the nanofiller. 

### 3.1. Rheological Properties

**Figure 3:**
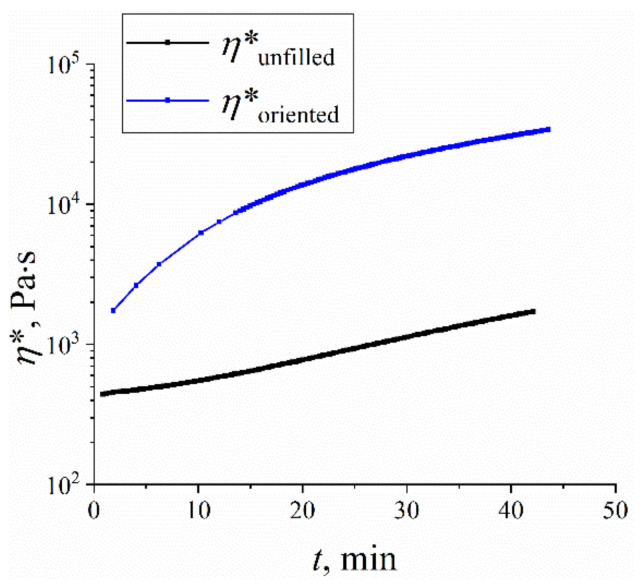
Experimental time dependence of the complex viscosity of unfilled R-BAPB (*η*^∗^_unfilled_) and R-BAPB filled by 0.1% of SWCNT (*η*^∗^_oriented_) at temperature 633 K. The error bars are equal to the instrumental error.

The rheological properties of the R-BAPB samples reinforced by SWCNT were investigated using experimental techniques and computer simulations. Analysis of the change in the rheological properties of R-BAPB at 633 K showed a slight increase in viscosity over time, Figure 3. The viscosity initially of the PI is 420 Pa s, and after 30 min the viscosity reaches 840 Pa s. The effect of increasing melt viscosity over time is attributed by many authors [55,56,82] to the cross-linking/branching processes of the polymer chain. However, in accordance with the experimental data, we did not observe any cross-linking processes or changes in the chemical structure of the polymer after a melt viscosity study. It is well known [83] that all PIs have strong intermolecular interactions and in particular R-BAPB [39,40,41]. In this connection, the increase in viscosity is probably associated with the process of structuring macromolecules of the R-BAPB in time. The incorporation of SWCNT into the PI leads to a significant increase in the viscosity of the system, from 1700 Pa s to 10,700 Pa s (after holding for 30 min). Probably, the presence of the SWCNT particles accelerates the process of ordering the R-BAPB molecules, leading to a significant increase in the system’s viscosity, Figure 3. 

This strong increase in viscosity in the melt might result in the R-BAPB chains’ structural ordering relative to the surface of a carbon nanotube that affecting the anisotropy of the rheological properties of an SWCNT-filled PI melt. To confirm the hypothesis above and to figure out the molecular mechanisms that determine the increased R-BAPB viscosity in the melt, we carried out a computer simulation of the cyclic deformation of the R-BAPB samples. The deformation was performed in different planes along (XZ, and YZ) and perpendicular (XY) to the SWCNT axis directed along the Z-axis. This simulation allows us to determine how the rheological properties of the R-BAPB interface layer depend on the orientation of the polymer chains of R-BAPB relative to the SWCNT surface. 

**Figure 4:**
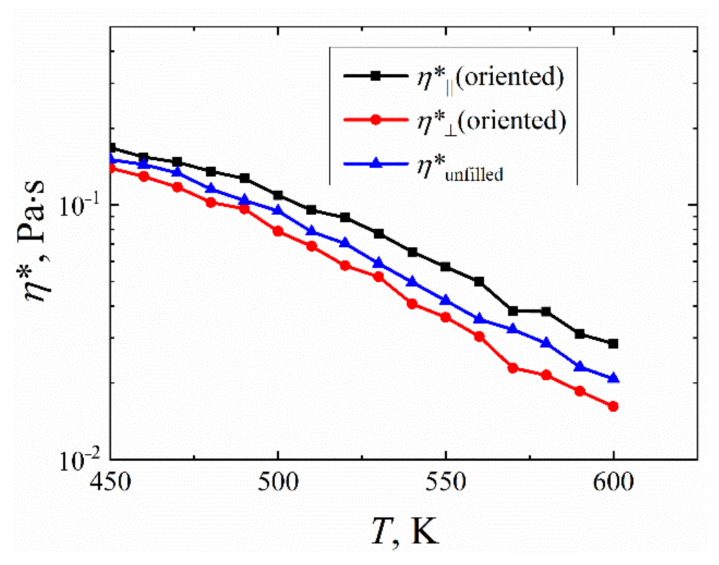
Temperature dependence of the complex viscosity *η*^∗^ of R-BAPB samples oriented by SWCNT. The data calculated along *η*^∗^_||_ (the results averaged for *η*^∗^ calculated in XZ and YZ shear directions) and perpendicular *η*^∗^_⊥_ (*η*^∗^ calculated in XY shear direction) to the SWCNT axis. Additionally, data for the unfilled amorphous R-BAPB samples *η*^∗^_unfilled_ (averaged over three directions of shear deformation) are presented. The rheological properties of the unfilled amorphous R-BAPB sample *η*^∗^_unfilled_ were taken for comparison from our previous study [75]. The error bars are compared to the size of the symbols. The results were obtained by computer simulations using Equations (1) and (2).

The rheological properties of the interfacial layer of PI R-BAPB in the vicinity of SWCNT were investigated at various temperatures. Using the method of cyclic deformation [76,77], the temperature dependences of the main rheological characteristics (elastic *G*^′^ and loss *G*^″^ moduli and complex viscosity *η*^∗^) along (XZ and YZ shear directions) and perpendicularly (XY shear direction) the SWCNT axis with deformation frequency *ω* = 1.74 × 10^9^ Hz were calculated, Figure 4 and Figure S6 in the Supplementary Information.

Because in an oriented sample the PI chains are predominantly located along the SWCNT direction (along the Z-axis), the orientation of the chains significantly affects the rheological characteristics of the oriented sample precisely during shear deformation along the SWCNT axis *η*^∗^_||_ (in XZ or YZ planes), Figure 4 and Figure S6. These rheological characteristics have higher values both compared with the values obtained upon shear deformation in the plane perpendicular to the SWCNT axis and in comparison with the average characteristics of an unfilled amorphous sample. Interestingly, the temperature dependence *η*^∗^(*T*), calculated for the deformation in the plane perpendicular to the SWCNT axis, is even lower than *η*^∗(^*T*) for the amorphous unfilled sample R-BAPB, Figure 4. This worsening of the rheological properties perpendicularly to the SWCNT surface might also be caused by weak structural ordering in the considered direction.

**Figure 5:**
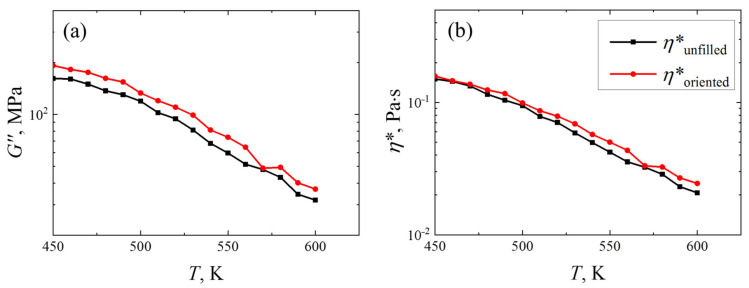
Temperature dependence of (**a**) loss modulus *G*^″^ and (**b**) complex viscosity *η*^∗^ of unfilled amorphous (*η*^∗^_*unfilled*_) and R-BAPB samples oriented by SWCNT (*η*^∗^_*oriented*_). The rheological properties of amorphous unfilled R-BAPB samples were taken for comparison from a previous study [75]. The error bars are compared to the size of the symbols. The results were obtained by computer simulations using Equations (1) and (2).

The temperature dependence of the elastic modulus *G*^′^ (see Figure S7 in the Supplementary Information) does not differ significantly for the amorphous and oriented R-BAPB samples, in contrast to the temperature dependence of the loss modulus *G*^″^ and complex viscosity *η*^∗^, Figure 5. The comparison of the temperature dependence of the loss modulus *G*^″^ and the complex viscosity *η*^∗^, presented in Figure 5, allows us to conclude that the addition of a carbon nanofiller into the polymer matrix of R-BAPB increases the loss modulus of the R-BAPB sample comparable to the values of the loss modulus *G*^″^ of the amorphous unfilled R-BAPB sample over the entire temperature range. Differences between the *η*^∗^(*T*) dependence of the R-BAPB sample oriented by SWCNT and the amorphous unfilled R-BAPB sample begin to appear only at temperatures above *T_g_* (~470 K [70]). Such a difference between the *η*^∗^ at high temperatures for an amorphous unfilled R-BAPB sample and an ordered one near the nanofiller surface may be associated with the interplanar interaction between the PI fragments, which are oriented along the nanotube surface.

The increase in the complex viscosity of the composite in the experiment appears to be greater than in the simulation. However, one should note that in the experiment, due to the experimental setup, we performed deformation of a sample in the direction of probable polymer chain orientation. Thus, the increase in complex viscosity in the direction parallel to the nanotube compared to that of the unfilled amorphous R-BAPB sample should be taken into account to compare the simulation results with the experimental ones. With this in mind, we can clearly conclude that the addition of SWCNT to R-BAPB leads to an increase in the complex viscosity of composites observed in both the experiment and the computer simulations. The computer simulation shows that the viscosity is much higher when the deformation direction coincides with the orientation of the polymer chains in the sample. Thus, the computer simulation results provide clear evidence that the viscosity increase observed in the experiment is related to polymer chain orientation in the composite, rather than the formation of interchain linkages. 

### 3.2. Mechanical Properties 

As the rheological properties of the SWCNT-filled nanocomposite are strongly influenced by the orientation of PI chains, the question arises about how the mechanical properties of the nanocomposite will depend on this orientation after cooling down the melt. Thus, to investigate this problem the mechanical properties of nanocomposites were examined by means of computer simulation and an experimental study.

**Figure 6:**
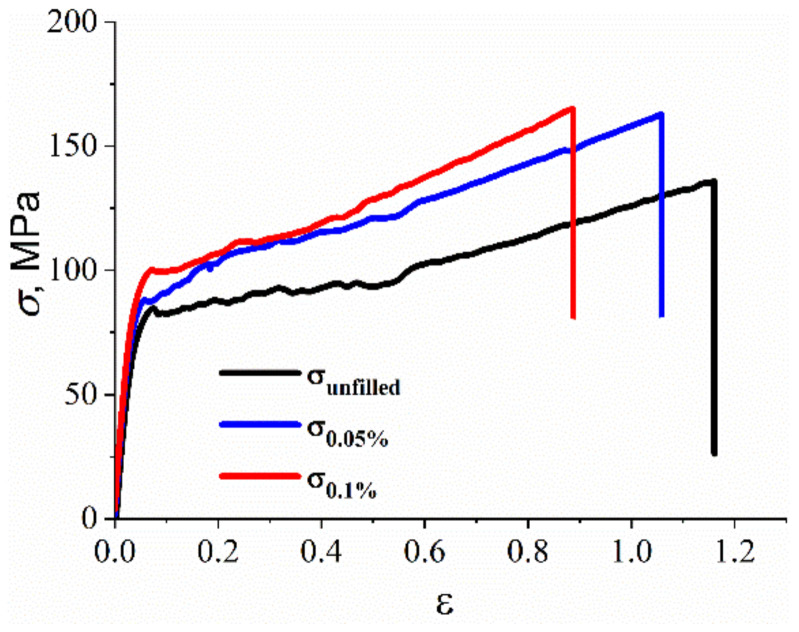
Experimentally obtained representative tensile stress-strain curve of R-BAPB fibers with carbon nanoparticles in the amorphous state for unfilled R-BAPB (*σ*_un_) and R-BAPB reinforced by SWCNT with 0.05% ($\sigma_{0.05\%}^{}$) and ($\sigma_{0.1\%}^{}$)—0.1% of nanofiller.

Samples of R-BAPB-based nanocomposite fibers reinforced by SWCNTs were experimentally deformed at a constant loading rate to study their mechanical properties at room temperature, Figure 6. 

From the stress-strain dependence presented in Figure 6, the values of tensile strength, Young’s modulus, and deformation at break were measured for both unfilled R-BAPB and SWCNT with R-BAPB fiber nanocomposites, Table 1. 

Although the difference in the obtained values of the mechanical properties of the composites is rather small compared to the properties of the unfilled polymer, we still see a qualitative increase in the composite mechanical properties of the composite when introducing carbon nanotubes into R-BAPB. The incorporation of SWCNT results in an increase in strength up to ~160 MPa and Young’s modulus up to ~3.12 GPa of R-BAPB fibers with 0.1% SWCNT mass fraction compared with an unfilled PI sample, Figure 6. The mechanical data collected are consistent not only with prior reported work [61] but also with other high-aspect-ratio materials such as the aforementioned FRCs [84] or CNCs [85]. The Young’s modulus of the first example ranges from 5.5 GPa to 10.5 GPa depending on the type of the reinforcing fiber, whereas CNCs demonstrate Young’s modulus ranging from 10.9 GPa to 9.2 GPa with relative humidity levels ranging from 15 to 95 percent. This demonstrates the significant potential of SWCNTs coated with R-BAPB for applications comparable to those of the aforementioned materials (food packaging, dentistry components, or surface-based polymer-polymer interaction biosensors). 

**Figure 7:**
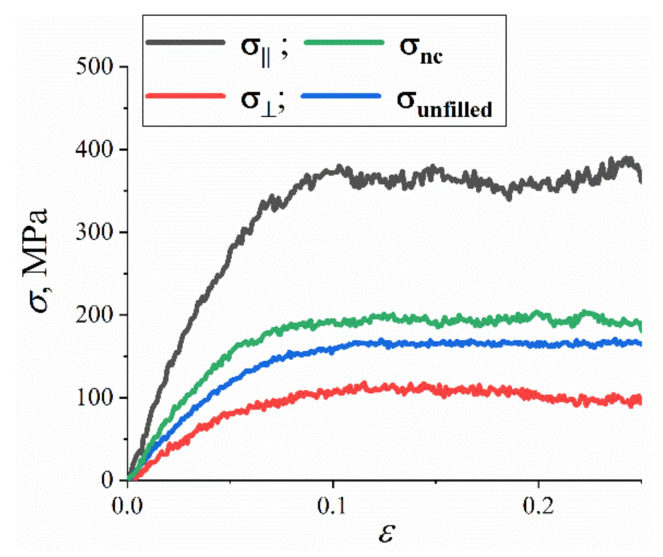
The representative stress-strain dependence for the unfilled amorphous R-BAPB and R-BAPB ordered by SWCNT. The stress-strain dependence $\sigma_{⊥}$ perpendicularly to the SWCNT was calculated as averaged by the X and Y directions, the stress-strain dependence along *σ*_||_ the SWCNT surface was in Z direction. Additionally, the averaged stress-strain dependence *σ*_nc_ of nanocomposites is shown. The averaging was carried out over three directions of applied deformations. The mechanical properties of amorphous unfilled *σ*_unfilled_ R-BAPB samples were taken for comparison with our previous study [41]. The results were calculated by computer simulations using Equation (3).

The anisotropy of the mechanical properties of nanocomposites might cause an increase in the values of the mechanical characteristics of R-BAPB-based nanocomposites upon the addition of SWCNTs near the surface of the carbon nanofiller. Using a similar approach to our previous study [41], a computer simulation of the mechanical properties of the PI R-BAPB near the SWCNT surface was performed. As for infinite graphene [41], the influence of the ordering of polymer chains on the mechanical properties change of polyimide interfaces was studied. For this, cooled samples of amorphous unfilled R-BAPB and R-BAPB oriented by SWCNT were deformed at room temperature with a strain rate of γ_d_ ≈ 1.8 × 10^8^ s^−1^. The results obtained showed that, as in the case of systems composed of graphene [41], the anisotropy of the mechanical properties of the R-BAPB sample oriented by SWCNT is also revealed. In the Z direction, along which the SWCNT axis is directed, the yield peak *σ_y_* and Young’s modulus *E* were three times higher than the *σ_y_* in the *X* and *Y* directions, which is perpendicular to the SWCNT axis, Figure 7.

The results obtained for the R-BAPB sample oriented by SWCNT were averaged over the three directions of deformation (X, Y, and Z) and compared with the previously obtained data [41] for amorphous non-oriented and unfilled samples of R-BAPB, Figure 7. 

Analysis of the calculated data showed that the dependence *σ*(*ε*) for the R-BAPB samples oriented by SWCNT lies higher than that dependence for the amorphous unfilled R-BAPB sample, Figure 7 and Table 2. In this case, the average values of Young’s modulus and yield peak of the sample oriented by SWCNT are greater than these characteristics of the amorphous unfilled sample, Table 2. 

The use in the computer simulation of the composite with infinite long SWCNT gives the opportunity to investigate through the computer simulations the mechanical properties of the polyimide nanocomposites more similarly to experimental studies. The greater increase in the mechanical characteristics of nanocomposites compared to the amorphous unfilled samples in the simulation versus the experiment might be caused by the fact that all carbon nanofiller particles in the experiment are not oriented toward deformation, in contrast to the simulation where the SWCNT is oriented along one of the deformation directions, Table 1 and Table 2. For the first time, we showed that using the infinite-long SWCNT oriented along one of the directions of deformation might lead to the anisotropy of the mechanical characteristics of the R-BAPB samples. Furthermore, the results obtained are in good qualitative agreement with the previous results of the mechanical property simulations for two crystallizable BPDA-P3 and ODPA-P3 in the presence of a nanofiller [43], where the structural ordering of the PI chains was also shown to lead to a similarly strong increase in the mechanical characteristics of PI-SWCNT-based nanocomposites in the glassy state. However, its impact on rheological properties was not studied before.

The results of computer simulation and experimental measurements are in good quantitative agreement with each other. In the simulation, the anisotropy of the mechanical characteristics of the R-BAPB chains ordered by SWCNT is observed, which most likely increases the average Young’s modulus and yield stress of the ordered R-BAPB sample compared to the results of the unfilled R-BAPB ones. 

## 4. Conclusions

In our previous computer simulations [39,40,41,42,43], the strong structural ordering of thermoplastic semi-crystalline polyimides near the carbon nanofiller can enhance its mechanical properties. In the current study, the microsecond-scale molecular dynamics computer simulation and comprehensive experimental work on the rheological and mechanical properties of nanocomposites based on thermoplastic PI R-BAPB reinforced with an SWCNT were performed. For the first time in the experiment, an interesting effect of a dramatic increase in the change rate of viscosity of R-BAPB in the melt over time in the presence of carbon nanotubes was obtained, resulting in a certain enhancement in the mechanical properties of the R-BAPB. On the basis of the experiments performed, it was hypothesized that the increase in the melt viscosity of polyimide melts with carbon nanotubes is mainly related not to the formation of a percolation mesh of carbon nanoparticles, but to the structural ordering of macromolecules near the nanotube surface. A computer simulation of the R-BAPB-based nanocomposites proved this result. Ordering of the polymer near the nanotube surface leads to the growth of the complex viscosity *η*^∗^ of composite that is higher than that for the amorphous unfilled R-BAPB sample both in the experiment and in a computer simulation. The new experimental data obtained on the R-BAPB-based nanocomposites filled with SWCNT compared extensively with the simulation results, confirming the idea of the influence of polyimide chain ordering near the carbon nanofiller on the mechanical characteristics of the composite material.

The strong anisotropy of rheological and mechanical characteristics of the R-BAPB-based nanocomposite was found. The increase in the viscosity of the polymer in the viscous-flow state and an increase in the values of the mechanical characteristics (Young’s modulus and yield peak) of the SWCNT-R-BAPB nanocomposites in the glassy state are stronger in the directions along the ordering of polymer chains close to the carbon nanofiller surface. The effect of the rheological property changes leading to the enhancement of the mechanical characteristics that were found for the R-BAPB samples might be applicable for other crystallizable polymers and materials based on them. The obtained results might be useful for the modern industry in the creation of new insulating coatings based on thermoplastic crystallizable heterocyclic polymers with improved performance properties.

## Figures and Tables

**Table 1:** Mechanical properties of unfilled amorphous R-BAPB and SWCNT-R-BAPB nanocomposite of fibers with different SWCNT mass fractions in the amorphous state. The results were obtained using experimental techniques.

Sample	Strength *σ,* MPa	Deformation at Break *ε,* %	Young’s Modulus E, GPa
R-BAPB	138 ± 11	104 ± 13	2.82 ± 0.07
R-BAPB + 0.05% SWCNT	163 ± 15	90 ± 13	3.01 ± 0.36
R-BAPB + 0.1% SWCNT	160 ± 11	88 ± 22	3.12 ± 0.36

**Table 2:** Mechanical properties (values of Young’s modulus *E* and yield stress *σ_y_* values) ordered by SWCNT R-BAPB samples, calculated along and perpendicularly to the SWCNT axis. The results for unfilled amorphous R-BAPB samples were taken from the previous study [41] for comparison. The results were obtained by computer simulations using Equation (4).

Direction of Applying Deformation	*E*, GPa	*σ_y_*, MPa
Along the SWCNT	2.1 ± 0.3	110 ± 10
Perpendicularly to the SWCNT	7.0 ± 0.2	380 ± 13
Average values for R-BAPB sample ordered by SWCNT	3.7 ± 0.2	200 ± 6
Average values for unfilled amorphous sample [41]	2.7 ± 0.3	169 ± 4

## Data Availability

The data presented in this study are available on request from the corresponding author. The data are not publicly available due to the large size of simulation trajectories and experimental datasets.

## Supplementary Materials

The following supporting information can be downloaded at: https://www.mdpi.com/article/10.3390/polym14153154/s1. **Figure S1.** Time dependence of the average (a) end-to-end distance (H_end-to-end_), (b) radius of gyration (R_g_), (c) ratio between (H_e-e_/R_g_)^2^, (d) asphericity (b), (e) acylindricity (c), and (f) relative shape anisotropy (κ^2^) for R-BAPB during simulation procedure. The error bars are compared to the size of the symbols. **Figure S2.** Time dependence of the nematic order parameter *S_N_* for polymer chains of R-BAPB in PI-SWCNT nanocomposites. The results were obtained using computer simulations. The error bars are compared to the size of the symbols. **Figure S3.** The chemical structure of the considered thermoplastic R-BAPB repeating unit. Arrows mark the vectors ***DP*** and ***PPI*** aligned along the phenylene and phthalimide planar moieties of PI R-BAPB correspondingly, for which we investigated the orientation along the nanotube axis. **Figure S4.** The dependence of the R-BAPB order parameter for the vector ***PPI*** and ***DP*** on the distance from the SWCNT axis. The results were obtained using computer simulations. The error bars are compared to the size of the symbols. **Figure S5.** The distribution of orientation angle *θ* of (a) ***DP*** and (b) ***PPI*** vectors directed along the planar moieties in PI R-BAPB-based nanocomposites with SWCNT after 9 μs of computer simulation. The results were obtained using computer simulations. **Figure S6.** Temperature dependences of (a) elastic modulus *G*′ and (b) loss modulus *G*″ of R-BAPB samples ordered by SWCNT. We calculated the data along (||) (*XZ* and *YZ* shear directions) and perpendicularly (⊥) (XY shear direction) to the SWCNT direction and an unfilled amorphous R-BAPB sample (averaged by three directions of shear deformation). The error bars are comparable to the symbol sizes. The rheological properties of amorphous unfilled R-BAPB samples were taken for comparison with a previous study. The results were calculated using computer simulations. The error bars are compared to the size of the symbols. **Figure S7.** Temperature dependences of the elastic modulus *G*′ of amorphous unfilled and R-BAPB samples oriented by SWCNT. The error bars are comparable to the symbol sizes. The rheological properties of the unfilled amorphous R-BAPB samples were taken for comparison from a previous study. We calculated the results using computer simulations. The error bars are compared to the size of the symbols.
