# Supplementary material for: Rheological and Mechanical Properties of Thermoplastic Crystallizable Polyimide-Based Nanocomposites Filled with Carbon Nanotubes: Computer Simulations and Experiments
Source: Polymers (Basel). 2022 Aug 2;14(15):3154. doi: 10.3390/polym14153154 (PMC9370852; doi:10.3390/polym14153154)
Supplement: Supplementary file 1 [file polymers-14-03154-s001.zip › polymers-1819170-supplementary.pdf]

Supplementary Information

Rheological and Mechanical Properties of Thermoplastic Crystallizable Polyimide-Based Nanocomposites Filled with Carbon Nanotubes: Computer Simulations and Experiments

Victor M. Nazarychev, Gleb V. Vaganov, Sergey V. Larin*,
Andrey L. Didenko, Vladimir Yu. Elokhovskiy,
Valentin M. Svetlichnyi, Vladimir E. Yudin, and Sergey V. Lyulin

Institute of Macromolecular Compounds, Russian Academy of Sciences (IMC RAS), Bolshoi pr. 31 (V.O.), St. Petersburg, 199004, Russia
*Corresponding author: selarin@macro.ru
tel: +7 (812) 3230216

S1. The generation of the system initial configurations in molecular dynamics.

The 10 μs-long computer simulation was carried out to generate in the computer simulation the R-BAPB polymer chains in the partial ordered configuration in the nanocomposite. The details of the previously developed generation techniques for polymer nanocomposites could be found in our previous studies. In particular, at a temperature of 600 K, which is about 100 degrees higher than the experimental value of glass transition temperature of the R-BAPB, a short (~ 10 ns) compression of the initial configuration in *X* and *Y* directions of the system was performed, during which the pressure increased to 300 bar. Immediately after compression, the 10 μs-long computer simulations were carried out. Namely, the sample was simulated for 1 μs at T = 600 K. To increase the translational mobility of the polymer chains of the R-BAPB sample, it was decided to increase the temperature by 100 degrees. Therefore, to accelerate the translation mobility of polymer chains in the system, the temperature was instantly increased to 700 K, and the simulation continued for 3.5 μs. Then the temperature was again lowered to 600 K. At this temperature, the simulation was performed for 5.5 μs, during which the average sizes of the polymer chains remained constant. So, the overall simulation time was 10 μs. Upon the simulation time, polymer chain gyration radius R_g, end-to-end distance $H_{end\text{-}to\text{-}end}$ and the shape parameters of the polymer chains (asphericity **b**, acylindricity **c**, and shape anisotropy, $\boldsymbol{\kappa}^2$) also calculated, Figure S1. The analysis of the corresponding time dependences of confirmed that the equilibrium state of the systems considered was reached and the R-BAPB samples obtained was used as initial state for the cooling procedure and the further calculation of the rheological and mechanical properties.

The value of the relative shape anisotropy $\boldsymbol{\kappa}^2$, asphericity **b**, and acylidricity **c** of the R-BAPB polymer chains were calculated using the relations

$$\kappa^2 = \frac{b^2 + (3/4)c^2}{R_g{}^4},$$
$$b = R_{g,x}{}^2 - 1/2\left(R_{g,z}{}^2 + R_{g,y}{}^2\right), \quad \text{(S1)}$$
$$c = R_{g,y}{}^2 - R_{g,z}{}^2.$$

where $R_{g,x}, R_{g,y}, R_{g,z}$ are the three main components of the tensor of inertia of polymer chains, correspondingly.

Size and shape parameters of R-BAPB chains allow us to conclude that they are in conformation of a coil deformed in a certain direction as the shape of the chains is characterized by relatively high asphericity values and low acylindricity. At the same time R_g and $H_{end\text{-}to\text{-}end}$ are slightly higher than that predicter theoretically.

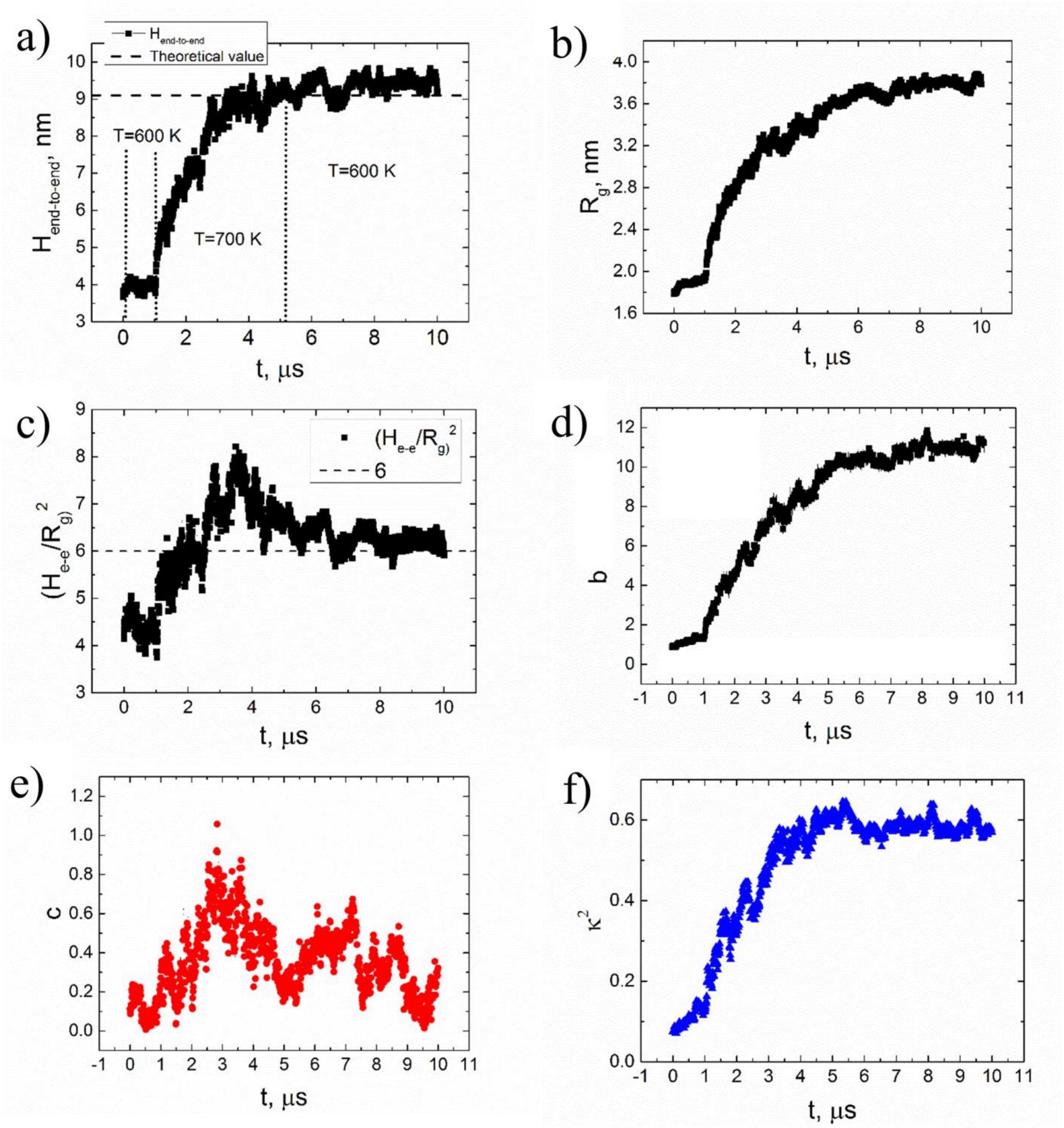

Figure S1. Time dependence of the average (a) end-to-end distance (**$H_{end\text{-}to\text{-}end}$**), (b) radius of gyration (**R_g**), (c) ratio between (**$H_{e\text{-}e}/R_g$**)^2, (d) asphericity (**b**), (e) acylindricity (**c**), and (f) relative shape anisotropy (κ^2) for R-BAPB during simulation procedure. The error bars are compared to the size of the symbols.

S2. The analysis of structural ordering of R-BAPB polymer chains near SWCNT.

Similarly to our previous studies, the structural ordering of R-BAPB near SWCNT surface was also observed in a computer simulation. For understanding the structure change of the polymer chains upon incorporation of the carbon nanofiller, we calculated the nematic order parameter S_N, which is the largest eigenvalue of the following order tensor:

$$Q_{\alpha\beta} = \frac{1}{N_{ch}} \sum_{i=1}^{N_{ch}} \frac{3}{2} \bar{u}_{i\alpha} \bar{u}_{i\beta} - \frac{1}{2} \delta_{\alpha\beta}, \quad \text{(S2)}$$

where N_{ch} is the number of chains for which the calculation is performed, $\bar{u}_i$ is the unit vector parallel to the end-to-end vector of ith chain, δ is the Kronecker delta, and α, β = *x*, *y*, or *z*, Figure S2.

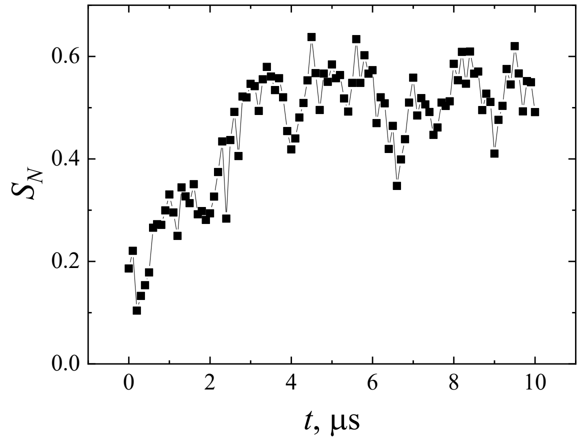

Figure S2. Time dependence of the nematic order parameter S_N for polymer chains of R-BAPB in PI-SWCNT nanocomposites. The results were obtained using computer simulations. The error bars are compared to the size of the symbols.

We can distinguish two parts on the time dependence of the nematic order parameter during 10 μs of simulation. In the first section (from 0 to 5 μs of simulation), the polymer chains are initially in a collapsed state after the generation of nanocomposite samples

(size of R-BAPB chains is much lower than experimentally predicted values and asphericity **b** is close to zero), and they unfold over time. During first 5 μs of simulation the average values of the distance between the ends and the radius of gyration reach constant values, see Fig. S1. In the second time interval (from 5 to 10 μs), the value of the nematic order parameter fluctuates around an average value (~ 0.5).

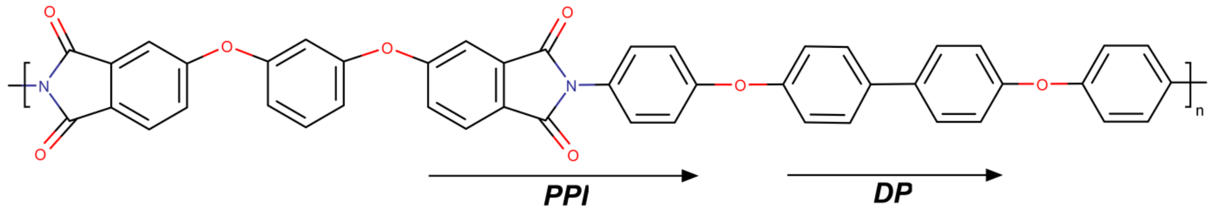

Figure S3. The chemical structure of the considered thermoplastic R-BAPB repeating unit. Arrows mark the vectors ***DP*** and ***PPI*** aligned along the phenylene and phthalimide planar moieties of PI R-BAPB correspondingly, for which we investigated the orientation along the nanotube axis.

Further, as in our previous papers in order to study the structure of the polymer-SWCNT interface, we analyzed the orientation of the phenylene and phthalimide planar moieties in the PI chains, represented by the ***PPI*** and ***DP*** vectors, correspondingly, see their definition Figure S3, and calculated the order parameters, *S(r)*

$$S(r) = \tfrac{3}{2}\left\langle \cos^2\theta(r) \right\rangle - \tfrac{1}{2}, \qquad \text{(S3)}$$

where r is the distance from the nanotube axis to the polymer chain planar moiety and θ is the angle between the vector ***PPI*** or ***DP*** directed along the planar moiety of the PI chains and the carbon nanotube axis, Figure S3.

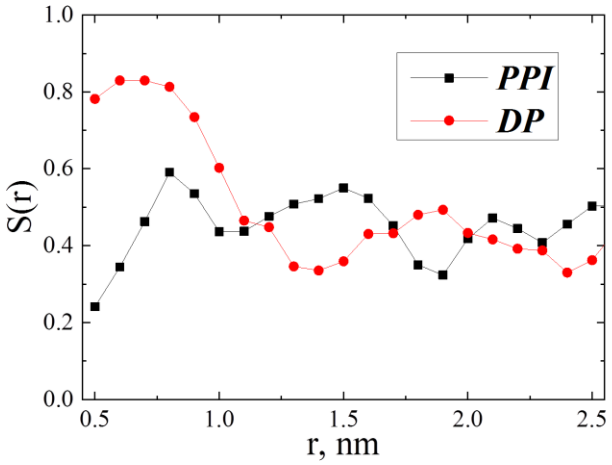

Figure S4. The dependence of the R-BAPB order parameter for the vector ***PPI*** and ***DP*** on the distance from the SWCNT axis. The results were obtained using computer simulations. The error bars are compared to the size of the symbols.

The dependence exhibits a few maxima corresponding to the close proximity to SWCNT surface polymer layers. In this case, the planar fragments of the R-BAPB polymer chains are oriented along the SWCNT surface, as evidenced by the values of the order parameter *S(r)* ~ 0.8 and ~ 0.75 near the SWCNT surface. These orientations correspond to the ~ 10° and ~ 20° angle between the SWCNT axis and flat vectors. The ***DP*** vector directed along the planar moiety in the diamine fragment exhibits a higher-order relative SWCNT axis compared to the ***PPI*** vector. Also, the distributions of angle θ between the SWCNT direction and planar moieties of PI R-BAPB (which were marked in Figure S3 by the ***PPI*** and ***DP*** vectors) have been calculated, Figs. S5. We observed the maximum on the distributions of angle θ at a distance of ~ 0.75 nm: therefore, the flat fragments are oriented to a greater extent along the SWCNT axis, Figs. S5.

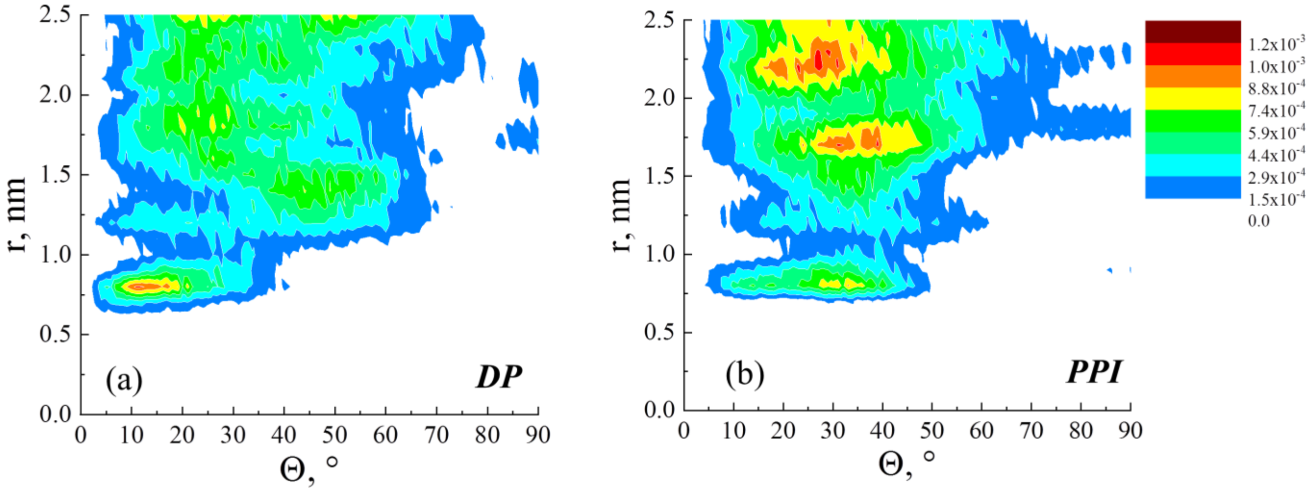

Figure S5. The distribution of orientation angle θ of (a) ***DP*** and (b) ***PPI*** vectors directed along the planar moieties in PI R-BAPB-based nanocomposites with SWCNT after 9 μs of computer simulation. The results were obtained using computer simulations.

S3. The study of rheological properties of R-BAPB polymer chains near SWCNT.

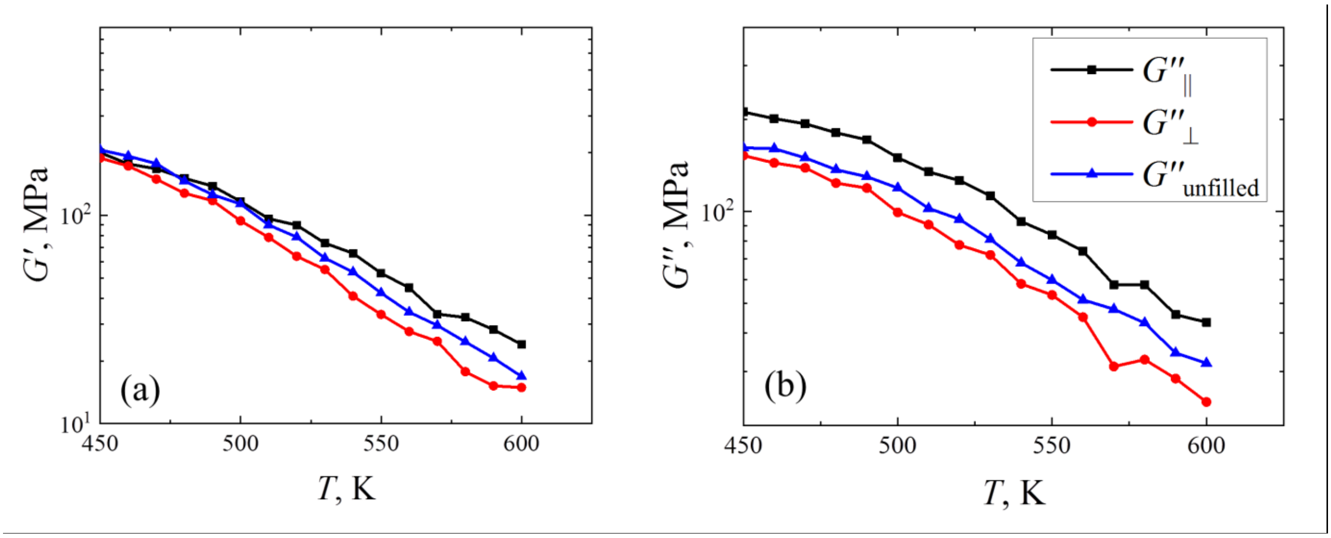

Figure S6. Temperature dependences of (a) elastic modulus G' and (b) loss modulus G'' of R-BAPB samples ordered by SWCNT. We calculated the data along (||) (*XZ* and *YZ* shear directions) and perpendicularly ($\perp$) (*XY* shear direction) to the SWCNT direction and an unfilled amorphous R-BAPB sample (averaged by three directions of shear deformation). The error bars are comparable to the symbol sizes. The rheological properties of amorphous unfilled R-BAPB samples were taken for comparison with a previous study. The results were calculated using computer simulations. The error bars are compared to the size of the symbols.

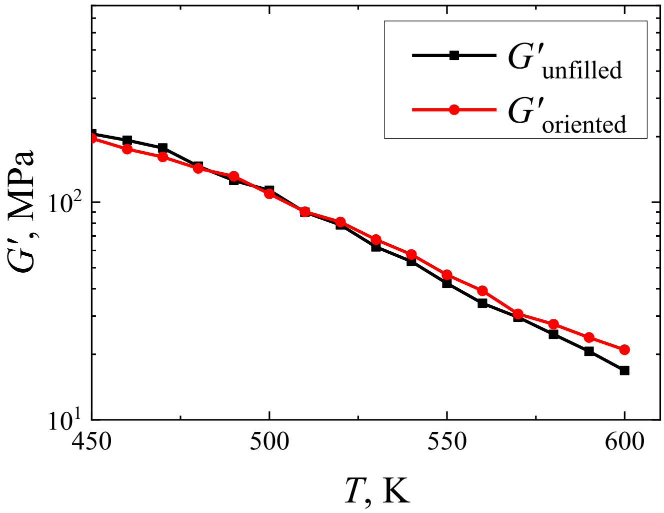

Figure S7. Temperature dependences of the elastic modulus G' of amorphous unfilled and R-BAPB samples oriented by SWCNT. The error bars are comparable to the symbol sizes. The rheological properties of the unfilled amorphous R-BAPB samples were taken for comparison from a previous study. We calculated the results using computer simulations. The error bars are compared to the size of the symbols.
